# Photoinitiated Polymerization of Cationic Acrylamide in Aqueous Solution: Synthesis, Characterization, and Sludge Dewatering Performance

**DOI:** 10.1155/2014/465151

**Published:** 2014-02-05

**Authors:** Huaili Zheng, Yi Liao, Meizhen Zheng, Chuanjun Zhu, Fangying Ji, Jiangya Ma, Wei Fan

**Affiliations:** ^1^Key Laboratory of the Three Gorges Reservoir Region's Eco-Environment, State Ministry of Education, Chongqing University, Chongqing 400045, China; ^2^Department of Architectural Engineering, Sichuan University of Science and Engineering, Zigong 643000, China; ^3^China National Offshore Oil Corporation (CNOOC), Tianjin Chemical Research and Design Institute, Tianjin 300131, China

## Abstract

A copolymer of acrylamide (AM) with acryloyloxyethyl trimethyl ammonium chloride (DAC) as the cationic monomer was synthesized under the irradiation of high-pressure mercury lamp with 2,2-azobis(2-amidinopropane) dihydrochloride (V-50) as the photoinitiator. The compositions of the photoinduced copolymer were characterized by Fourier transform infrared spectra (FTIR), ultraviolet spectra (UV), and scanning electron microscope (SEM). The effects of 6 important factors, that is, photo-initiators concentration, monomers concentration, CO(NH_2_)_2_ (urea) concentrations, pH value, mass ratio of AM to DAC, and irradiation time on the molecular weight and dissolving time, were investigated. The optimal reaction conditions were that the photo-initiators concentration was 0.3%, monomers concentration was 30 wt.%, irradiation time was 60 min, urea concentration was 0.4%, pH value was 5.0, and mass ratio of AM to DAC was 6 : 4. Its flocculation properties were evaluated with activated sludge using jar test. The zeta potential of supernatant at different cationic monomer contents was simultaneously measured. The results demonstrated the superiority of the copolymer over the commercial polyacrylamide as a flocculant.

## 1. Introduction

The fast industrialization, rapid urbanization, and unrestrained use and exploitation of natural resources throughout the world have increased the water pollution by different types of pollutants [1, 2]. Treatment of the contaminated water generates large quantities of sludge as the negative by-product [3, 4]. Sludge treatments are based on both the stabilization processes to block or reduce biological activity and on dewatering processes to reduce sludge volume [5]. Generally, operating cost for sludge treatment can take approximately 50% of the total operating cost of the whole waste water treatment plant [3, 6], among which 30–50% is in relation to the sludge dewatering [5]. Hence, it is necessary to improve water removal efficiency of the sludge so that the cost of sludge transportation and handling can be reduced as far as possible [7].

Generally speaking, moisture content of typical sewage sludge was up to 97%–99% [8, 9]. Usually sludge dewatering is difficult even at high pressure, considering the colloidal and compressible nature of sludge caused by the presence of organic components, mainly bacterial cells and EPS (extracellular polymeric substances) [7, 10]. Researches indicated that adding chemical conditioners such as flocculants and coagulants can help the sludge particles agglomerate into larger particles or flocs prior to solid–water separation [11]. Because sludge is negatively charged, the flocculants and coagulants with positive charge are commonly used for this purpose [10, 12].

As a kind of organic flocculant, cationic polyacrylamide (CPAM) can have the effect of charge neutralization and absorption bridge in sludge dewatering [13]. It has been widely used in sludge dewatering due to its outstanding advantages such as low dosage demand, high efficiency, good solubility, and being pollution free [14]. The flocculation performance of CPAM flocculants primarily depends on the molecular weight and charge density [15]. Furthermore, the level of molecular weight and charge density of CPAM depend significantly on the type and amount of cationic monomers involved in the process of synthesis. Acryloyloxyethyl trimethyl ammonium chloride (DAC) seems to be one of the most commonly used cationic commoner. Compared with other frequently used cationic monomers such as methacryloyloxyethyl trimethyl ammonium chloride (DMC) and dimethyldiallylammonium chloride (DMDAAC), DAC has its own unique advantages. Because there is one less hydrophobic methyl group in the carbon chain of DAC than that of DMC, the hydrophilic and flexibility of the copolymer of acrylamide (AM) and DAC(P(AM-DAC)) should be better than those of the copolymer of AM and DMC(P(AM-DMC)). In addition, P(AM-DAC) has higher cationic degree and more reasonable cationic structure distribution than P(AM-DMDAAC) because the reactivity of DAC is significantly higher than that of DMDAAC. Thus, in this work DAC was selected as the cationic monomer to synthesize with AM to prepare copolymer with molecular weight and cationic degree as high as possible by testing and making decision on the optimal reaction conditions.

CPAM can be prepared by copolymerization of AM with cationic monomers. In general, the most widely used polymerization reaction for AM and cationic monomer is free radical copolymerization in aqueous solution. It can be initiated by heat [16], microwave [17, 18], rays [19], and ultraviolet (UV) [20]. In contrast with other methods, photoinitiated polymerization has various advantages. It can be carried out at low reaction temperature. Low temperature is beneficial for the production of polymer with high molecular weight by avoiding the side effect of branching and cross-linking, which can be commonly encountered at high temperature [20–22]. Meanwhile, the photopolymerization is easy to operate and control, which requires only simple equipment, economized energy sources, making it an eco-friendly process [21]. In addition, photoinduced polymerization is believed to have the ability to make surface modification on surface graft polymerizations [23, 24]. However, so far the most common method to induce polymerization of AM and cationic monomers is by heat. There have been some reports on the synthesis of P(AM-DAC) in aqueous solution induced by heat [25, 26], but there are few reports currently on the synthesis induced by UV light. The existing studies about the polymerization initiated by UV light are mostly concerned with the microstructure and reaction kinetics of resulting cationic copolymers [27], and other similar polymerization of P(AM-DAC) was carried out under the irradiation of metal halide lamps or low pressure mercury lamp [28, 29] Considering the low efficiency of low pressure mercury lamp and the high price of metal halide lamp, we used high pressure mercury lamp to achieve high initiating efficiency and cost saving. Besides, the report on flocculation performance of the polymer induced by UV light is rarely mentioned; therefore, we evaluated the sludge dewatering performance of the copolymer induced by UV light compared with a commercial PAM.

In this paper, synthesis of the cationic polyacrylamide P(AM-DAC) was investigated using a novel initiation system, photoirradiation. 2,2′-Azobis(2-amidinopropane) dihydrochloride (V-50) was introduced into the system as the photoinitiator for the polymerization of acrylamide with DAC as the cationic monomer in aqueous solutions. The photoinitiator can initiate polymerization at low temperature because the polymerization with photoinitiator does not have any dependencies on reaction temperature. Simultaneously, the effects of the photoinitiators concentration, monomers concentration, CO(NH_2_)_2_(urea) concentration, pH value, mass ratio of AM to DAC, irradiation time on the molecular weight, and dissolving time were investigated intensively. Study of the characterizations of the photoinitiated polymer P(AM-DAC) was realized by using such methods as Fourier transform infrared spectra (FTIR), ultraviolet spectra (UV), and scanning electron microscope (SEM). Finally, sludge dewatering experiment was carried out to evaluate the dewatering efficiencies of the resulting polymers and a commercially available PAM. Meanwhile the influences of molecular weight and cationic monomer content of the resulting polymers on the sludge dewatering performance were investigated.

## 2. Experimental

### 2.1. Materials

The monomer AM was industrial material and used without further purification, sourced from Chongqing Lanjie Tap Water Company (Chongqing, China); acryloyloxyethyl trimethyl ammonium chloride (DAC) (80 wt.%) was provided by Guangchuangjing Import and Export Co., Ltd, Shanghai, China; EDTA (AR) and urea (AR) were purchased from Chongqing Chuan-dong Chemical Co., Ltd. (Chongqing, China); Photoinitiator 2,2′-azobis(2-amidino-propane) dihydrochloride (V-50) was purchased from Ruihong biological technology Co., Ltd. (Shanghai, China), and other reagents were analytical grade, used as received. Commercial PAM was bought from Guanghui Chemical (Dalian, China) (molecular weight provided by supplier was 500 g·mol^−1^, measured by our Lab was 460 g·mol^−1^). Deionized water was used throughout this work. High purity nitrogen was used in the synthesis of the polymer in order to drive away the air existing in the reactor.

### 2.2. Synthesis of the Copolymer

**Figure 1:**
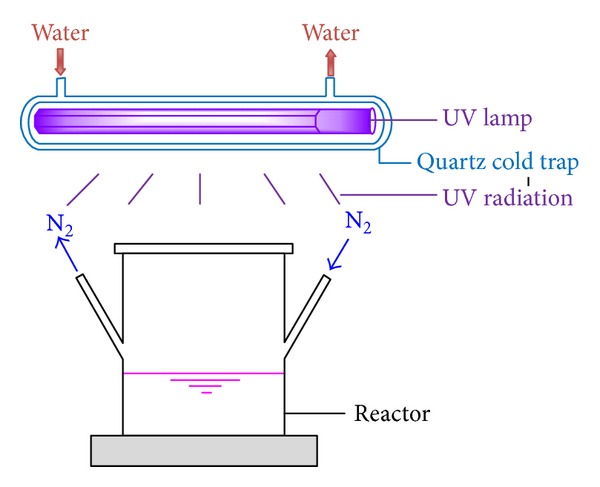
Photoinitiated reaction device.

**Scheme 1:**
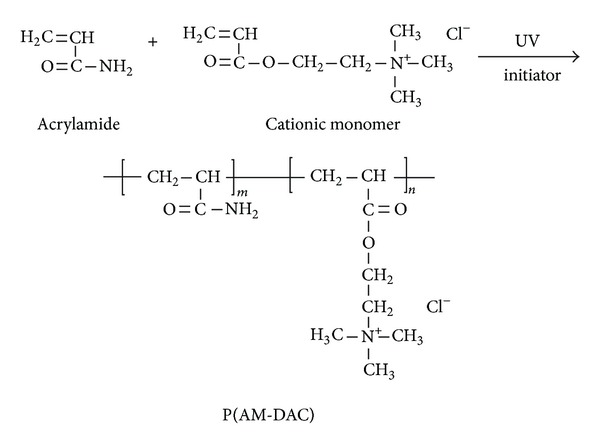
Proposed reaction scheme of the synthesis.

P(AM-DAC) was synthesized from two monomers, namely, AM and DAC, in an aqueous reaction system. The reaction took place under UV irradiation, and the specific procedure was shown step by step as follows. AM and DAC of varying dosages were dissolved in deionized water in the reaction vessel. The mixture was stirred with a glass rod until the monomers dissolved completely. Next, a certain dosage of urea was added into the vessel. The pH of the aqueous solution was adjusted to designated values with 0.1 M sodium hydroxide and 0.1 M hydrochloric acid. Purified nitrogen was bubbled through the aqueous solution at room temperature for about 20 min to remove the oxygen. Then, the photoinitiators were added into the solution. The reaction vessel was sealed and transferred to the photoinitiated reaction device (Figure 1), the core of which is a 500-W high pressure mercury lamp. The irradiation time under UV lamp was set from 25 min to 120 min in a series of experiments. The proposed reaction scheme for polymerization was shown in Scheme 1. After irradiation, the photopolymerized products were removed from the reaction vessel and then purified with acetone and ethanol, followed by the process, of drying, breaking, and packaging.

### 2.3. Characterization

The Fourier transform infrared Spectra (FTIR) of the cationic polyacrylamide was measured by the 550 Series II infrared spectrometer (Mettler Toledo Instruments Co., Ltd., Switzerland); the ultraviolet (UV) spectra of the cationic copolymer were detected by the TU - 1901 double beam UV-visible light spectrophotometer (Beijing Puxi General Instrument Co., Ltd., China). In addition, after pretreatment with spray gold, morphology of the product was determined using VEGAII LMU scanning electron microscopy (SEM) (TES-CAN Co., Ltd. of Chech), which was compared with that of the commercially available PAM.

### 2.4. Measurement of Molecular Weight

The intrinsic viscosity ([*η*]) of the photoinduced polymer was determined in 1 M NaCl aqueous solution with an Ubbelohde viscometer at 30 ± 0.05°C. The average molecular weight (Mw) of the copolymer was calculated with the Mark-Houwink equation as follows [20]:

(1) $$\begin{array}{c} \text{Mw}={\left\{\frac{10000\times [\eta ]}{3.73}\right\}}^{1/0.66}. \end{array}$$

### 2.5. Measurement of Dissolving Time

The dissolved time of the copolymer was measured using the conductance method. The specific steps were as follows: 100 mL of distilled water was added to a 100 mL beaker as the reaction vessel, which was placed in the water bath with magnetic stirring device. Then the stir was started and the electrode of conductivity meter was inserted into the beaker while the temperature of the water bath was controlled at 30 ± 1°C for 20 min. After that about 0.038~0.042 ± 0.001 g of the sample was gradually blended in the distilled water. If the conductance value did not change in 3 minutes, the sample was considered completely dissolved. Finally, the time required for the complete dissolution of the sample was recorded.

### 2.6. Sludge Dewatering Experiment

To investigate the influences of such conditions as molecular weight and cationic monomer content of the resulting polymers on the sludge dewatering performance and evaluate the dewatering efficiencies of the photoinitiated copolymers and a commercially available PAM, a program-controlled Jar-test apparatus (ZR4-6, Zhongrun Water Industry Technology Development Co. Ltd., China) was used for sludge dewatering experiments. The sludge samples were obtained from the Dadukou Wastewater Treatment Plant located in Chongqing, China. All sludge samples were placed in 2.5-L polyethylene tanks and kept in the refrigerator at 4°C after being transferred to the laboratory. All experiments were carried out no later than three days after taking sludge samples to minimize possible changes in sludge properties during storage. The sludge was characterized by a moisture content of 98.6%, mass density of 1.002 kg*·*L^−1^, and pH value of 7.3. 500 mL of sludge was transferred into beakers and then different dosage of flocculant was added. The stirring rate was 100 rpm continuing for 30 seconds to attain the complete mix of the flocculant with sludge, followed by a 30 seconds slow stirring period at 50 rpm to promote floc growth. Then, the conditioned sludge was poured into a Buchner funnel for filtering under a vacuum pressure of 0.09 MPa for 30 min or until the vacuum could not be maintained (in <30 min). The filterability of the sludge was measured by the filter cake moisture content (FCMC):

(2) $$\begin{array}{c} \text{FCMC}\%=\frac{M_{e}-M_{d}}{M_{e}}\times 100\%, \end{array}$$

where *M*_*e*_ was the weight of filter cake at the end of filtration and *M*_*d*_ was the weight of filter cake after drying at 105°C.

*Measurement Method for Zeta Potential*. A certain quantity of sludge supernate taken after dewatering was used for the detection of zeta potential to evaluate the charge neutralization effect of the resulting polymers with analogous molecular weight and various contents of cationic monomer. The electrophoretic mobility of these samples was measured and then converted to the zeta potential value at room temperature using Malvern zetasizer Nano ZS90 (Malvern instruments Ltd., UK).

## 3. Results and Discussion

### 3.1. Characterization of Copolymer

**Figure 2:**
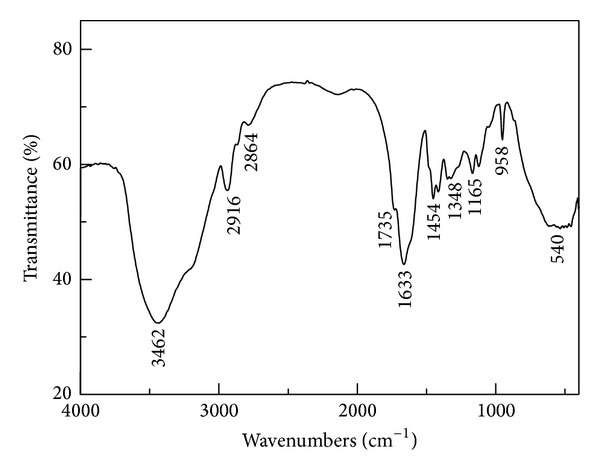
FTIR spectra of the copolymer. (Mass ratio of AM to DAC was 6 : 4; average molecular weight of the photoinitiated copolymer was 600 × 10^4^; purified with ethanol and acetone for many times before measurement.)

Figure 2 was the FTIR spectra of the copolymer. The characteristic absorption bands at 3462 cm^−1^ and 1633 cm^−1^ were assigned to the stretching vibration of –NH_2_ and –C=O of –CONH_2_ groups in the AM unit. The characteristic absorption peaks at 2916 cm^−1^ and 2864 cm^−1^ came from the –CH_3_ and –CH_2_ asymmetry stretching vibration. The characteristic absorption peak at 958 cm^−1^ arose from N^+^ (CH_3_)_3_ stretching vibration of DAC unit. The characteristic absorption peak at 1454 cm^−1^ was due to deformation vibration absorption of methylene group. The characteristic absorption bands at 1735 cm^−1^ and 1165 cm^−1^ were owe to the stretching vibration of C=O and C–O in the DAC unit, respectively. All the above mentioned characteristic peaks were evidences that the two monomers were copolymerized.

**Figure 3:**
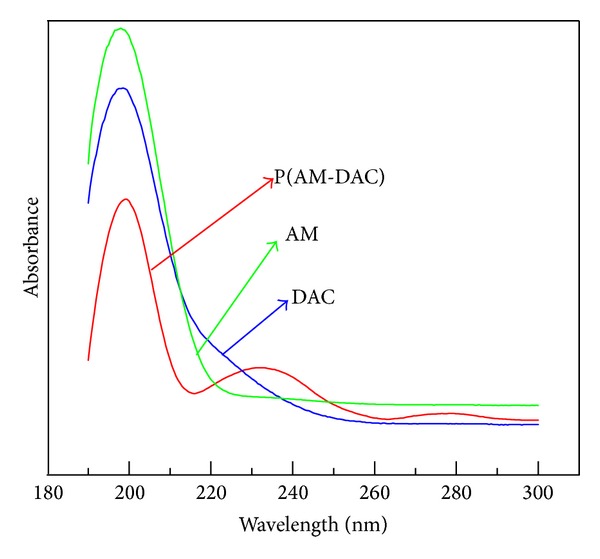
UV spectra of (a) P(AM-DAC). (Mass ratio of AM to DAC was 6 : 4; average molecular weight of the photoinitiated copolymer was 600 × 10^4^) (b) acrylamide, and (c) DAC.

The UV spectra of the photoinitiated copolymer P(AM-DAC), acrylamide, and DAC were presented in Figure 3 as the curves (a), (b), and (c), respectively. Because the feed concentration of photoinitiators was very slow and the copolymer was purified with acetone and ethanol for many times, the concentration of residual photoinitiator would be much lower. Thus, the UV absorbance of the photoinitiator that remained in copolymer could be ignored as there was no obvious UV absorbance in the range of 190–300 nm in our test. It was observed that there were strong absorption peaks at 198.5 nm and 198 nm for monomer AM and DAC, respectively, which was consistent with the report that the absorption peak of UV spectra of *α*, *β*-unsaturated amides with no substitute for their N atoms was at around 200 nm [30]. From Figure 3(a), we also found that there were three strong absorption peaks at about 200 nm, 233 nm, and 280 nm for the photoinitiated copolymer. The peak at 200 nm probably resulted from the residual AM in the completed reaction, while the new absorption peak at 233 nm and 280 nm proved the generation of a new material in the resulting polymer, which was an evidence that the copolymerization of AM and DAC took place under the irradiation of UV lamp.

**Figure 4:**
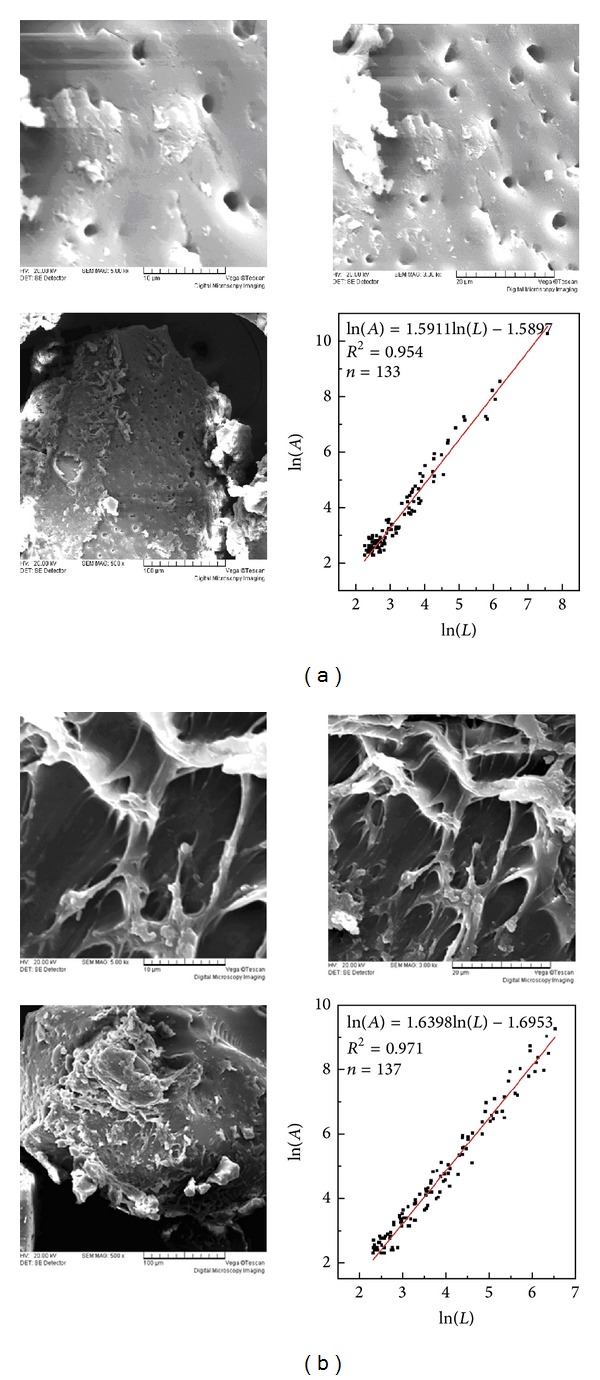
SEM micrographs of (a) commercial PAM and (b) photoinitiated P(AM-DAC). (Mass ratio of AM to DAC was 6 : 4; average molecular weight of photoinitiated-copolymer was 600 × 10^4^; purified with ethanol and acetone for many times before measurement.)

Figure 4 showed the surface morphology of commercially available PAM and photoinitiated P(AM-DAC). As shown in Figure 4, close cross-linking and gel network structure were observed in the photoinitiated P(AM-DAC), whereas the commercial PAM showed a comparatively looser and flatter surface. The difference in morphological structure may result from the introduction of DAC and some kind of surface modification induced by ultraviolet light, which was probably similar to existing related reports [21, 23, 24]. The net structure of photo-induced copolymer was favorable for the penetration of water into the polymeric network, so that the solubility of product could be improved [31]. Meanwhile, the net structure was conducive for the improvement of the ability of adsorption and bridging of P(AM-DAC), and more colloidal particles, organic pollutants in water could be adsorbed accordingly. Additionally, the average fractal dimension, which was the slope of the fitted straight line to Ln(A) (area) as a function of Ln(L) (perimeter), was calculated using Image-pro Plus 6.0 Software to quantitatively estimate the surface morphology of samples [32]. The results showed that the fractal dimensions of commercial PAM and photoinitiated P(AM-DAC) were 1.5911 and 1.6398, respectively.

### 3.2. Effect of Photoinitiators Concentration

**Figure 5:**
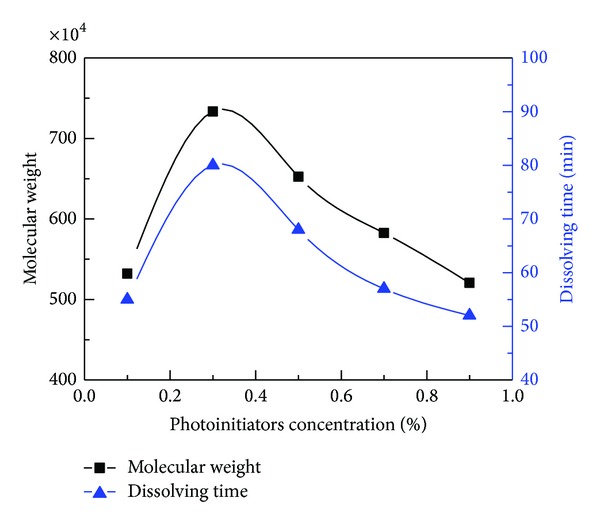
Effect of photoinitiators concentration on the molecular weight and dissolving time. (Polymerization conditions: monomers concentration was 30 wt.%, mass ratio of AM to DAC was 6 : 4, urea concentration was 0.4%, pH value was 5.0, and irradiation time was 60 min.)

As is shown by Figure 5, the molecular weight increased as the initiators concentration rose from 0.1% to 0.3% and decreased with further increase in the initiators concentration. This phenomenon could be explained by the cage effect and primary radical termination with stable large molecules. When the photoinitiators concentration was relatively low, cage effect may lead to low polymer yields and low average molecular weights due to predominating termination of the growing chains inside the cage as there were fewer monomer units to contribute propagation [33, 34]. As the initiator increased from 0.1% to 0.3%, more and more primary radicals were generated and more and more of them could escape from their “cages” to react with monomers, leading to continuing growth of the molecular weight in this process. However, further increase in initiators concentration above 0.3% might increase the termination and chain transfer rate, which led to the decrease of molecular weight. This trend accorded with the classical kinetic theory, which predicts that the kinetic chain length depends on the square root of the initiator concentration, as also indicated by most of the previous works [35, 36]. In addition, because of the light absorption of initiator and scattering effect in reaction medium, the light intensity decreased along the radiation path. This variance would lead to the spatially inhomogeneous distribution of free radicals in the system, so that the conversion rate and molecular weight of various layers in the reaction vessel were different. This was different from the heat-induced polymerization, which was characterized with uniform active center distribution and reaction speed. It had been reported that polymer with ultrahigh molecular weight was readily prepared in photo-induced polymerization due to the graded distribution of light intensity within reaction vessel [37]. Besides, Figure 5 showed that the change of dissolving time was consistent with the change of molecular weight, which indicated that dissolving time relied mainly on the molecular weight if there was little cross-linking in the process of polymerization. Therefore, the favorable value of the initiators concentration was 0.3%.

### 3.3. Effect of Monomers Concentration

**Figure 6:**
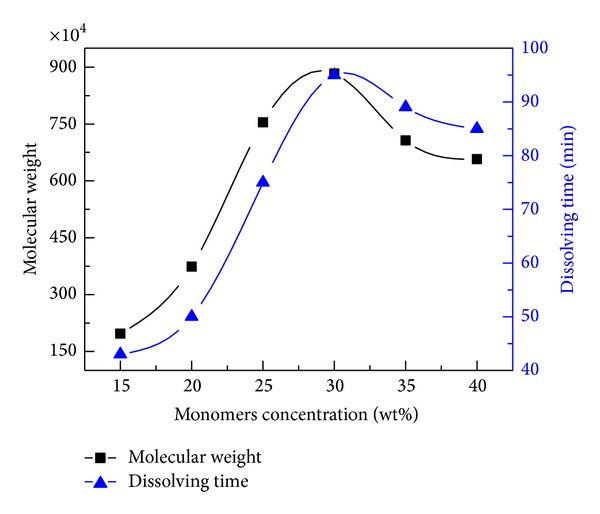
Effect of monomers concentration on the molecular weight and dissolving time. (Polymerization conditions: photoinitiators concentration was 0.3%, mass ratio of AM to DAC was 6 : 4, urea concentration was 0.4%, pH value was 5.0, and irradiation time was 60 min.)

As shown in Figure 6, the molecular weight and dissolving time increased at the beginning and then decreased with increasing monomers concentration. The initial monomers concentration was important partly because the free radical number was determined by the monomers concentration at the start of the reaction [20]. When the monomers concentration was quite low, the production rate of the free radicals was so slow that a low free radical concentration existed. These free radicals might be more prone to premature termination because of the cage effect, as previously mentioned [38]. In addition, there was comparatively less possibility of contact and collision between monomers of AM and DAC at low monomers concentration, which was not conducive to the growth of the molecular chain. Thus, low molecular weight was typical at low monomers concentration. As the monomers concentration increased from 15 wt.% to 30 wt.%, a sharp increase in molecular weight was observed. This trend could be explained as the consequences of weakened cage effect and increased probability of contact and collision between monomers at increasing monomers concentration. This process indicated that photo-induced polymerization could still be accounted for by the general law of free radical polymerization; that is, the kinetic chain length is proportional to the monomers concentration. However, with the further increase in monomers concentration, the molecular weight decreased. This could be explained as follows: first, a larger generation of heat released by the highly exothermic reactions and the lower thermal diffusivity of the reacting mixture at relatively high monomers concentration led to temperature rise of reaction system, which would lower the degree of polymerization, and second, the higher viscosity of the system and the consequently low rate of diffusion-controlled termination at relatively high monomers concentration (i.e., the gel effect) caused uncontrollable reactions and low molecular weight [39, 40]. Moreover, the change of dissolving time was consistent with the change of molecular weight, and the trend could be explained by the fact that copolymer with high molecular weight dissolves more slowly in water. Therefore, 30 wt.% was adopted as the optimal concentration of monomers concentration.

### 3.4. Effect of Irradiation Time

**Figure 7:**
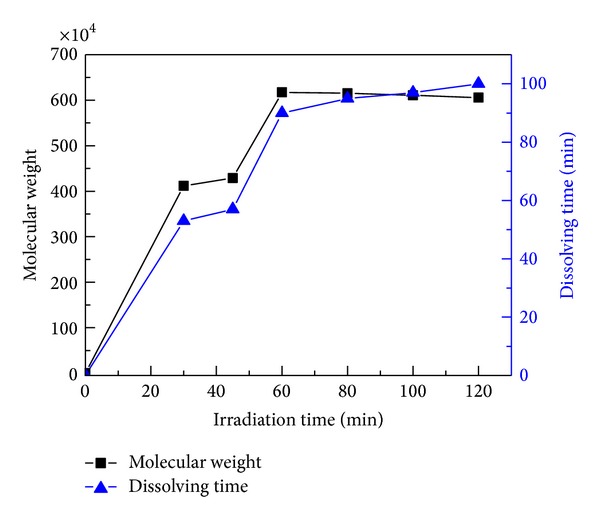
Effect of irradiation time on the molecular weight and dissolving time. (Polymerization conditions: photoinitiators concentration was 0.3%, monomers concentration was 30 wt.%, mass ratio of AM to DAC was 6 : 4, urea concentration was 0.4%, pH value was 5.0.)

It can be seen from Figure 7 that the molecular weight increased sharply at the beginning with the increase of irradiation time and kept constant after 60 min. It was consistent with the basic characteristics of free radical polymerization: slow initiation, fast propagation, and rapid termination. At the beginning of the reaction, the medium was transparent and the UV light could easily transfer through the medium, in which water-soluble photoinitiator could absorb the light and produced a large number of free radicals in a short time at the surface and subsurface of the medium and in turn initiate polymerization reaction between AM and cationic monomers. As the irradiation time increased, the transparent solution gradually turned into a gelatinous texture, leading to the weakened penetration of UV light through the reaction vessel. Besides that, the possibility of chain transfer reaction and disproportionation reaction increased as the irradiation time increased. As a result, there was no further increase of degree of polymerization in the late stage of polymerization, and molecular weight remained almost unchanged after reaching the maximum at 60 min accordingly. Some studies reported that the time taken to reach the maximum molecular weight of polyacrylamide in aqueous polymerization initiated by heat was over 5.0 h [41]. Obviously, it was much higher than the polymerization time initiated by UV light. In addition, similar results were also reported in dispersion polymerization [42]. In this sense, the polymerization initiated by UV light was more energy efficient and more effective than polymerization initiated by heat. Meanwhile, the dissolving time of the photoinitiated polymer increased all the time of the reaction. It may result from the high degree of crosslinking with longer reaction time. Consequently, 1.0 h was adopted as the optimal irradiation time.

### 3.5. Effect of Urea Concentration

**Figure 8:**
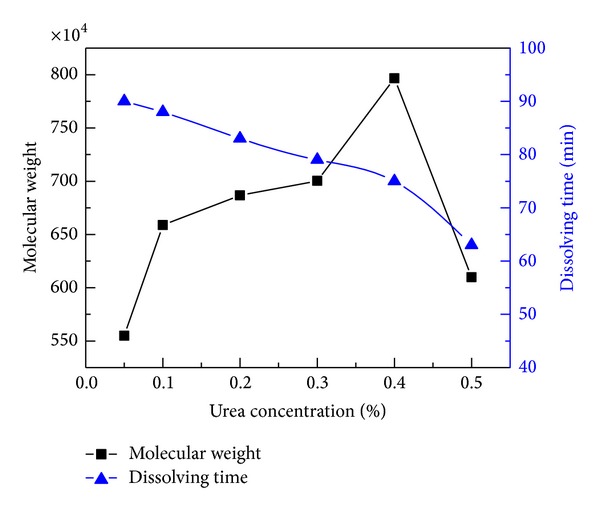
Effect of urea concentration on the molecular weight and dissolving time. (Polymerization conditions: photoinitiators concentration was 0.3%, monomers concentration was 30 wt.%, mass ratio of AM to DAC was 6 : 4, pH value was 5.0, and irradiation time was 60 min.)

In the actual applications, both high molecular weight and good solubility are the favorable characteristics of CPAM as flocculants. However, the dissolution rate of polymer is very slow because it is difficult for water to seep into the polymer due to the significant differences of size and velocity between water and polymer. According to the theory of instant dissolution, the dissolution rate of a polymer can be accelerated when it contained some micromolecules with a structure similar to the polymer. Because both acrylamide and urea contain amide group, urea is widely used in the synthesis of polyacrylamide to improve the dissolution rate of the polymer. Urea can weaken the hydrogen bonding of the molecular chain within CPAM because the active amide groups in urea will react with the amide group in polymer, which will cause the decrease of the intermolecular forces and the chance of crosslinking [9, 43].  Figure 8 showed that the molecular weight increased slowly at the urea concentration ranging from 0.05% to 0.4% and then decreased sharply at the urea concentration above 0.4%. The highest molecular weight of 800 × 10^4^ was obtained when the concentration of urea was equivalent to 0.4%. The changes of molecular weight can be interpreted as follow: besides a solubilizer, urea can take part in oxidation-reduction reactions as a reducing agent at low urea concentration, which would benefit the increase of kinetic chain length, and the molecular weight increased accordingly. However, with the further increase of urea concentration, the probability of chain transfer increased, which was not desired for preparing a polymer with high molecular weight [6]. In addition, excess urea may affect the quality of the product.  Figure 8 also showed that the dissolving time of CPAM continued to drop as the concentration of urea increased from 0.05% to 0.50% and the dissolving time of CPAM was 75 min at the maximum molecular weight. Based on the discussion, the optimal concentration of urea was 0.4%.

### 3.6. Effect of pH Value

**Figure 9:**
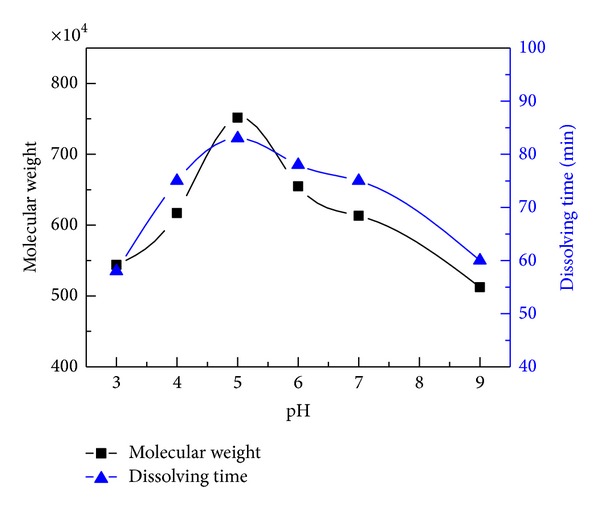
Effect of pH value on the molecular weight and dissolving time. (Polymerization conditions: photoinitiators concentration was 0.3%, monomers concentration was 30 wt.%, mass ratio of AM to DAC was 6 : 4, urea concentration was 0.4%, and irradiation time was 60 min.)

The effect of pH on molecular weight and dissolving time was shown in Figure 9. The pH value varied from 3 to 9 in this experiment. It was found that both the molecular weight and dissolving time increased firstly and then decreased. In strong acidic conditions, the intramolecular and intermolecular imidization reaction occurred easily due to the amide bond in acrylamide, resulting in the crosslinking between polymers, which is one of the most important reasons leading to low molecular weight and weak solubility of CPAM [27]. While under alkali conditions, a hydrolysis of CPAM would take place to produce NH_3_ or NH_3_·H_2_O, which will then react with acrylamide to form Nitrilotripropionamide [N(CH_2_CH_2_CONH_2_)_3_], NTP. NTP, as a reducing agent and chain transfer agent, would lead to polymerization termination or chain transfer with low molecular weight [44]. Moreover, the reaction solution would acquire increasing amounts of negative charges as the pH value increased, resulting in the increased electrostatic repulsion between radicals or the changes of mobility, flexibility, and configuration in the polymer solvents [45]. In addition, Figure 9 showed that the dissolving time was 83 min if the pH was 5. In general, weak acidic environment was ideal for the polymerization reaction and pH 5 was adopted as the optimal condition.

### 3.7. Effect of the Mass Ratio of AM to DAC

**Figure 10:**
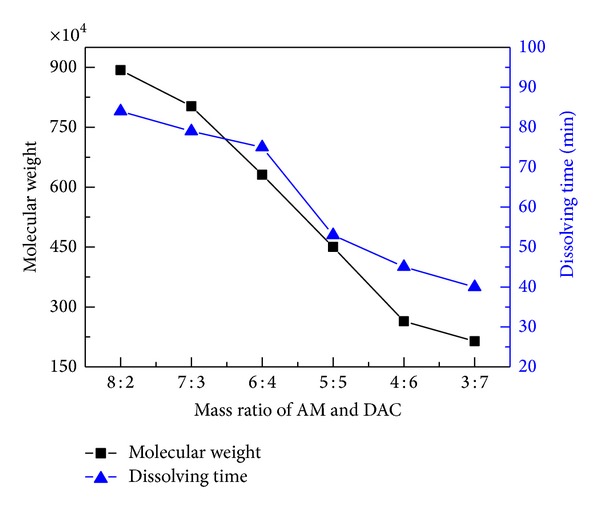
Effect of mass ratio of AM to DAC on the molecular weight and dissolving time. (Polymerization conditions: photoinitiators concentration was 0.3%, monomers concentration was 30 wt.%, urea mass concentration was 0.4%, pH value was 5.0, and irradiation time was 60 min.)

Compared with PAM, CPAM has not only the adsorption bridging effect but also charge neutralization effect on sludge dewatering due to the adding of cationic monomers to PAM. As shown in Figure 10, both molecular weight and the dissolving time decreased all the time as the mass ratio of AM to DAC decreased from 8 : 2 to 3 : 7. Prior research showed that the reactivity ratios of AM and DAC were 2.2864 and 0.3835, respectively, indicating that the reactivity of AM was higher than that of DAC [25]. Thus, copolymer with higher molecular weight could be prepared readily with higher dosage of AM. When the amount of DAC increased, the time required to polymerization increased due to the low reactivity of DAC, resulting in the increased time at high temperature. High temperature led to the increase of chain transfer rate constant of acrylamide in solution. In addition, cationic groups such as quaternary ammonium groups existing in DAC had space hindered effect, which will hamper the diffusion of monomer to the polymer [38]. So the molecular weight of copolymer decreased with the DAC increased. Furthermore, the decrease of dissolving time of CPAM with the increase of DAC content can be explained as follows: DAC is a quaternary ammonium salt and its monomer and homopolymer could dissolve in water readily [46]. After DAC was synthesized with AM, the hydrophilicity of PAM could be greatly improved. When combined with the subsequent sludge dewatering experiment, the optimal mass ratio of AM to DAC was 6 : 4 in this study.

### 3.8. Sludge Dewatering Performance

**Figure 11:**
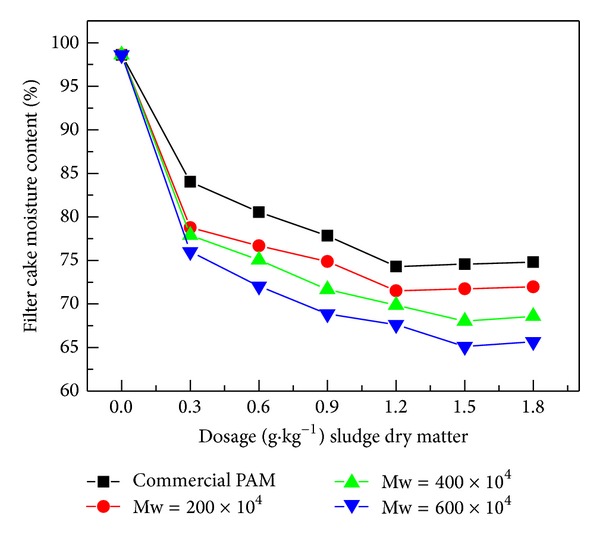
Contrast of dewatering efficiencies of several synthesized CPAMs and commercial PAM on filter cake moisture content at different dosages. (The mass ratio of AM to DAC of the synthesized CPAMs was 6 : 4; molecular weights of the synthesized CPAMs were 200, 400, and 600 × 10^4^ g·mol^−1^, respectively; the various molecular weights of the copolymers were obtained by adding various dosages of the photoinitiator; the concentration of polymer solution was 0.1%.)

In order to evaluate the flocculation effect of the copolymer prepared by the photoinitiated polymerization on sludge dewatering, the moisture content of the filter cake and zeta potential was measured.  Figure 11 showed the effects of photoinitiated P(AM-DAC) with same mass ratio (*m*_AM_ : *m*_DAC_ = 6 : 4) and different molecular weight (200, 400, 600 × 10^4^ g·mol^−1^) and commercial PAM on filter cake moisture content with various dosages. It indicated that the filter cake moisture content decreased firstly with the increase of flocculant dosage and increased slowly when the flocculant dosage above 1.5 g·kg^−1^ sludge dry matter. The lower filter cake moisture content could be achieved using the synthesized copolymer; thus, the synthesized CPAMs were more effective than the commercial PAM in sludge dewatering. In addition, for synthesized CPAMs, the lower filter cake moisture content could be achieved with the higher molecular weight polymer under the same dosage of flocculant. It indicated that the polymer with higher molecular weight had better absorption and bridge effect.

Table 1 listed the contrast results of sludge dewatering performances of several CPAMs with analogous molecular weight and various contents of DAC. Considering the absorption bridging effect of a polymer that depends mainly on the molecular weight of the polymer, keeping the molecular weight of the polymers analogous in this study could be helpful for us to diminish the differences in bridging effect of various polymers in order to evaluate the effect of cationic monomer content of polymer on sludge dewatering more accurately. As shown in the table, the zeta potential increased sharply until the value went above 0. A reverse of the net charge was observed with the increase of cationic monomer content in this experiment. Meanwhile, the moisture content of the filter cake decreased when the mass ratio changed from 10 : 0 to 6 : 4 and then increased. The phenomenon could be explained as follows: as the cationic monomer content increased, the electrostatic repulsion among the polymer chains increased, leading to the better stretching of the polymer chain, which was conducive to the enhancement of the bridging effect of the polymer on sludge particles. Besides, the effect of charge neutralization of the polymer on the sludge particles with negatively charge increased with the increasing cationic monomer content; thus, the surface charge of the sludge particles shifted gradually from negative values to zero as evidenced by the zeta potential data, resulting in the reduced electrostatic repulsive force among the sludge particles, which benefitted the sludge particles to agglomerate into larger particles or flocs [39]. When the mass ratio of DAC to AM increased to 5 : 5, the particle surface of sludge turned into positively charged and repulsion between particles increased; thus, the filter cake moisture content increased.

## 4. Conclusions

The photoinitiated copolymer of acrylamide with acryloyloxyethyl trimethyl ammonium chloride (DAC) as the cationic monomer was synthesized for sludge dewatering with 2,2-azobis(2-amidinopropane)dihydrochloride (V-50) as the photoinitiator. FTIR and UV spectra showed that the synthesized product was a copolymer of AM and DAC. SEM images provided the surface morphology of the copolymer.

The optimal reaction conditions for preparing photo-induced P(AM-DAC) were as follows: photoinitiators concentration was 0.3%, monomers concentration was 30 wt.%, the irradiation time was 60 min, urea concentration was 0.4%, pH value was 5.0, and the mass ratio of AM to DAC was 6 : 4.

The sludge dewatering experiment at various molecular weights and various cationic monomer contents of the photo-induced copolymer demonstrated the superiority of the copolymer product over PAM as a flocculant, and both absorption bridging and charge neutralization played important roles in the sludge dewatering process of the photo-induced copolymer.

## Figures and Tables

**Table 1:** Contrast of sludge dewatering performances of several CPAMs with analogous molecular weight and various contents of DAC.

Samples number	Mass ratio of AM to DAC	Molecular weight (×10^4^) g·mol^−1^	Zeta potential mV	Filter cake moisture content %
1	10 : 0	560	−18.9	77.8
2	8 : 2	550	−10.4	74.3
3	7 : 3	500	−5.6	70.2
4	6 : 4	530	−1.29	63.6
5	5 : 5	480	1.67	68.9

The analogous molecular weight of copolymers was attained by adding various dosages of the photo-initiator; the dosage of all samples was 1.5 g·kg^−1^ sludge dry matter; the concentration of polymer solution was 0.1%.
